# To Approach or Avoid Alcohol? Automatic and Self-Reported Motivational Tendencies in Alcohol Dependence

**DOI:** 10.1111/j.1530-0277.2011.01620.x

**Published:** 2012-02

**Authors:** Helen Barkby, Joanne M Dickson, Louise Roper, Matt Field

**Affiliations:** Division of Clinical Psychology, University of LiverpoolLiverpool, United Kingdom; Windsor Clinic, University Hospital AintreeLiverpool, United Kingdom; School of Psychology, University of LiverpoolLiverpool, United Kingdom

**Keywords:** Implicit Cognition, Ambivalence, Motivational Conflict

## Abstract

**Background:**

Motivational conflict is central to alcohol dependence, with patients reporting motivation to limit their drinking at the same time as urges to drink alcohol. In addition, dual process models of addiction emphasise the power of automatic cognitive processes, particularly automatic approach responses elicited by alcohol-related cues, as determinants of drinking behavior. We aimed to examine the strength of automatic and self-reported alcohol approach and avoidance tendencies among alcohol-dependent inpatients relative to matched controls.

**Methods:**

A total of 63 alcohol-dependent patients undergoing detoxification and 64 light-drinking controls completed a stimulus-response compatibility (SRC) task, which assesses the speed of categorization of alcohol-related pictures by making symbolic approach and avoidance movements. We also included modified versions of the SRC task to assess automatic motivational conflict, that is, strong approach and avoidance tendencies elicited simultaneously by alcohol-related cues.

**Results:**

There were no differences between alcohol-dependent patients and controls on the SRC task, although individual differences in the quantity of alcohol consumed before entering treatment were significantly positively correlated with the strength of approach (but not avoidance) tendencies elicited by alcohol-related cues. Automatic approach tendencies were also positively correlated with self-reported “approach” inclinations and negatively correlated with self-reported “avoidance” inclinations.

**Conclusions:**

Although alcohol-dependent patients and matched controls did not differ on automatic approach and avoidance tendencies elicited by alcohol-related cues, individual differences in the quantity of alcohol consumed before entering treatment were associated with the strength of automatic approach tendencies elicited by alcohol cues.

Addiction is characterized by a central paradox: the discrepancy between drug users’ expressed intentions (to abstain from drug use), and their behavior, which is characterized by repeated relapses and continued drug use (Heather, 1998; McCusker, 2001). The concept of motivational conflict is fundamental to understanding this paradox, with addiction recognized as a motivational problem involving conflict between inclinations to use drugs (“approach”) and inclinations to refrain from drug use (“avoidance”) (Heather, 1998; Orford, 2001). There is a long-standing distinction between an approach/appetitive motivational system that directs behavior toward positive or desirable events, and an avoidance/aversive motivational system that directs behavior away from negative or undesirable events (Davidson, 1993; Fowles, 1980; Gray, 1982; Lang et al., 1990). While these systems may be reciprocally activated, with high activity in one associated with low activity in the other, it has been demonstrated that the two are independent, dissociable, and can be activated simultaneously to elicit different motivational states (Cacioppo et al., 1999).

Motivational models of addiction incorporate this approach–avoidance distinction. In an extension of Cox and Klinger’s (1988) motivational model of alcohol use, Breiner and colleagues (1999) propose that there are 2 independent and opposing motivational pathways: approach inclinations to drink alcohol and avoidance inclinations against drinking alcohol, which give rise to an evaluative space that in turn determines the decision to drink or not drink. More recent models suggest that these motivational processes operate at multiple levels of awareness; thus, a person may or may not be aware of conflicting approach and avoidance inclinations (Cox et al., 2006).

There is an evolving focus toward the role of implicit or automatic cognitive processes in addiction. This requires a definitional distinction between “automatic” cognitive processes, which are spontaneous, fast, and sometimes occur outside of conscious awareness, and “controlled” cognitive processes, which are deliberate, slow, and require conscious awareness (Wiers et al., 2007). It has been argued that drug use leads to the development of automatic processes that promote approach behavior toward drug-related cues and ultimately drug-taking (Curtin et al., 2006; Robinson and Berridge, 1993; Tiffany, 1990). Among individuals attempting to limit their drug use, controlled processes are engaged to either inhibit or override these automatic approach tendencies and promote drug avoidance. These models therefore posit an approach–avoidance conflict between automatic appetitive responses to drug cues (“drink!”) versus controlled processes engaged in an attempt to promote abstinence (“don’t drink!”). With repeated experience of the negative consequences of drug use, automatic negative associations could also develop, leading to avoidance responses that are automatically elicited when the drug user encounters a drug-related cue (Wiers et al., 2006).

Evidence supports the existence of conflicting approach and avoidance inclinations that operate in both controlled and automatic processing. With regard to self-reports (controlled processes), qualitative coding of spontaneous reactions during alcohol cue exposure suggests that comments related to the desire to drink and the desire to limit drinking often occur together, and the relative approach–avoidance balance predicts the overall strength of craving (Smith-Hoerter et al., 2004). Validation studies of the Approach and Avoidance of Alcohol Questionnaire (AAAQ) (McEvoy et al., 2004) also support the approach–avoidance distinction in both dependent (Klein et al., 2007) and nondependent populations (Stritzke et al., 2007). Importantly, approach and avoidance inclinations are positively (rather than negatively) correlated which suggest that the two can occur simultaneously (McEvoy et al., 2004), and they predict unique variance in drinking variables among dependent and nondependent populations (Klein et al., 2007). Alcohol-dependent drinkers report both stronger approach and avoidance inclinations than controls, and among patients, approach–avoidance profiles distinguish “high lapsers” from “abstainers” and may longitudinally predict lapses (Stritzke et al., 2007). Therefore, among alcohol-dependent patients, self-reported approach and avoidance are separable, and their activation is not invariably reciprocal.

A separate body of evidence suggests an important role for automatic cognitive processes in heavy drinking. For example, attentional biases for alcohol-related cues are seen in heavy social drinkers and alcohol-dependent drinkers, but not light social drinkers (Field and Cox, 2008; Field et al., 2004), they are associated with craving strength (Field et al., 2009), and predictive of prospective alcohol use (Cox et al., 2002, 2007). Furthermore, there is evidence that extinction training to reduce attentional biases can lead to improved drinking outcomes in alcohol-dependent samples (Fadardi and Cox, 2009; Schoenmakers et al., 2010). Other studies have focused on automatic memory associations between alcohol-related cues and concepts related to approach and avoidance. Studies that used the Implicit Association Test (Greenwald et al., 1998) with social drinkers indicate that strong associations between approach-related concepts and alcohol-related concepts are correlated with several drinking variables, such as the frequency of binge drinking, even after controlling for self-report measures (Ostafin and Palfai, 2006; Palfai and Ostafin, 2003). We have used the stimulus-response compatibility (SRC) task (De Houwer et al., 2001) to measure behavioral approach tendencies elicited by alcohol-related cues. In the SRC task, participants are required to categorize substance-related and control pictures by moving a manikin figure either toward or away from the picture. Studies with smokers (Mogg et al., 2003) and cannabis users (Field et al., 2006) have demonstrated that substance users are faster to approach substance-related pictures and avoid control pictures than when they have to make the opposite response (i.e., avoid substance-related pictures and approach control pictures). This suggests that substance-related cues elicit automatic approach responses in substance users. In relation to alcohol use, studies have found that heavy but not light social drinkers show such a bias to approach rather than avoid alcohol-related pictures (Field et al., 2008, in press), and the magnitude of this bias is correlated with the strength of self-reported craving (Field et al., 2005, 2008). As with the work on attentional bias, recent work has shown that “retraining” these automatic approach responses to alcohol-related cues can influence drinking behavior (Wiers et al., 2010, 2011).

Currently, studies assessing automatic approach tendencies remain confined to nonclinical samples. However, a conflict between approach and avoidance may characterize attentional bias in treatment-seeking, alcohol-dependent samples. Alcohol-dependent patients show a bias to orient their attention toward alcohol-related pictures when those cues are presented very briefly, which perhaps indicates an automatized appetitive reaction; however, when those cues are presented for longer durations, patients show overt avoidance of the cues, which may reflect an aversive reaction that could be driven by either strategic or automatic processes (Noel et al., 2006; Stormark et al., 1997; Townshend and Duka, 2007). This provides preliminary evidence for motivational conflict in alcohol-dependent individuals. At present, it is unclear whether this conflict occurs within automatic processes (i.e., automatic alcohol-related cognitions may be simultaneously appetitive and aversive), or whether it represents a conflict between automatic attentional bias and strategically driven attentional avoidance. However, no previous studies have investigated this motivational conflict in other areas of automatic processing, such as automatic response tendencies. One issue with currently available tasks such as the SRC task is that they only provide an index of the strength of automatic approach responses that is *relative* to the strength of automatic avoidance responses: These tasks are unable to distinguish between individuals with weak approach and weak avoidance tendencies (indifference) and individuals with strong approach and strong avoidance (ambivalence). Therefore, the SRC task may require some modification if it is to measure independent approach and avoidance tendencies in alcohol-dependent individuals.

Our primary aim was to investigate motivational tendencies to approach and avoid alcohol at both controlled and automatic levels of processing in alcohol-dependent individuals, relative to a matched control group of light social drinkers. Our secondary aim was to examine the relationship between automatic and controlled approach and avoidance to determine the extent of coherence or conflict between them. Our participants completed a self-report measure of approach and avoidance tendencies (the AAAQ), together with a modified SRC task that permitted us to examine the strength of both automatic approach *and* avoidance responses elicited by alcohol cues.

Our primary hypotheses were that, compared to a control group, alcohol-dependent patients would: (i) self-report stronger approach and avoidance inclinations on the AAAQ and (ii) demonstrate stronger automatic approach and avoidance tendencies elicited by alcohol-related cues on the modified SRC task. We also investigated the relationships between self-reported and automatic measures of these motivational tendencies, although here our hypotheses were more exploratory. For example, it seems likely that self-reported approach inclinations would be positively associated with automatic cue approach (see Field et al., 2005, 2008), and self-reported avoidance inclinations would be positively correlated with automatic cue avoidance. On the other hand, if avoidance is primarily a strategic process that is engaged when participants become aware of automatic approach tendencies elicited by alcohol cues, then self-reported avoidance inclinations should be positively correlated with automatic cue approach.

## Materials and Methods

### Participants

#### Alcohol-Dependent Group

Sixty-three alcohol-dependent individuals nearing the end of inpatient alcohol detoxification were recruited from a specialist alcohol treatment unit in Liverpool, UK (49 male, 14 female, mean age = 45.25 years, SD = 8.89). Two participants (3%) were currently employed, and the remainder were either unemployed, retired, or on long-term sick leave. All were clinically assessed as alcohol-dependent prior to detoxification. Inpatient detoxification occurred on the unit ward, lasted an average of 5 days, and comprised medication and 24-hour nursing care. Eligible participants were approached during detoxification, after a minimum of 24 hours and once recovered from severe withdrawal symptoms. Participation occurred on the penultimate or final day of detoxification, when participants were alcohol-free, no longer experiencing withdrawal symptoms, and fit for discharge.

#### Nondependent Control Group

Sixty-four current light social drinkers were recruited from the local community (e.g., local adult further education colleges, church networks) (37 male, 27 female, mean age = 43.88 years, SD = 12.35). Fifty-seven (89%) were currently employed; the remainder were either unemployed, retired, or studying. We attempted to match the control and alcohol-dependent groups on age and socioeconomic status (the latter was based on current employment status and the age of leaving full time education). Unfortunately, we were unable to match the groups on employment status and education, as the control group tended to be better educated, and were more likely to be currently employed, than the alcohol-dependent group. Current light social drinking was defined as consumption of at least 1 alcoholic drink during the past month and weekly consumption under 10.5 and 7 units of alcohol per week for men and women, respectively. These criteria were chosen as they correspond to half of the maximum weekly intake of alcohol as recommended by the U.K. government (Edwards, 1996).

Exclusion criteria for both groups were as follows: (i) positive breath alcohol level, (ii) current dependence on other substances, (iii) significant medical illness, (iv) comorbid severe and enduring mental health disorder, and (v) overt cognitive impairment; and additionally for the nonclinical group: (vi) history of alcohol dependence. All participants spoke fluent English, had normal or corrected-to-normal vision, and provided written informed consent. The study was approved by the University, Local Research Ethics Committee, and NHS Trust Research Governance Committee.

### Clinical Assessment

Participants were administered a standard assessment battery prior to detoxification. This included a self-report index of recent alcohol consumption (Sobell et al., 1979) which yielded outcome variables of total number of drinking days, total weekly units consumed, and mean units consumed per drinking day. Severity of alcohol dependence, withdrawal, and self-reported mood were assessed with the Leeds Dependence Questionnaire (LDQ) (Raistrick et al., 1994), Windsor Clinic Alcohol Withdrawal Assessment Scale (WCAWAS) (Metcalfe et al., 1995), and Hospital Anxiety and Depression Scale (HADS) (Zigmond and Snaith, 1983), respectively. The LDQ has good internal consistency (α = 0.94), high test–retest reliability over a 2- to 5-day interval (*r* = 0.95), and concurrent validity because of its association with other measures of dependence (Raistrick et al., 1994). Interrater reliability for the WCAWAS is high (*r* = 0.84; Metcalfe et al., 1995), it is strongly associated with other self-report measures of withdrawal severity, and it is currently recommended for the assessment of withdrawal severity as part of routine clinical practice (Raistrick et al., 2006). The HADS has adequate internal consistency as indicated by inter-item correlations for anxiety and depression subscales (*r* = 0.41 to 0.76 and *r* = 0.30 to 0.60, respectively; Zigmond and Snaith, 1983).

### Materials: SRC Task Stimuli

Pictorial stimuli for the SRC task were 14 alcohol-related and 14 control (stationery-related) color photographs. Alcohol-related photographs varied by beverage type (e.g., beer, cider, wines, spirits) and setting (e.g., still life of beverage, liquor store, a model drinking). Each was paired with a stationery-related (control) photograph matched as closely as possible on perceptual characteristics (e.g., complexity, brightness, color) and structural content (e.g., size of object, person present, setting). Photographs were based on a previously validated set (Field et al., 2005, 2008) which were recreated and modified to represent the real-life drinking context of clients attending the clinic. All photographs were 93 mm high × 123 mm wide. The SRC task was programmed in Inquisit software (version 2.0; Millisecond Software, 2004) and presented on a laptop with a 14-inch monitor and standard keyboard, with 4 labeled response keys (up, down, left, right).

### Procedure

Eligible participants were invited to take part in a study investigating the relationship between cognitive processes and alcohol consumption. All participants were tested individually in private rooms either in the clinic (alcohol-dependent group) or in community colleges or church groups (control group). Participants were seated at a desk with the laptop monitor for the SRC task positioned 50 cm in front of them. Participants in the control group first completed a self-report questionnaire comprising questions about demographics, education, and health, together with the drinking diary, HADS, and the LDQ (this information was obtained from patient records from the alcohol-dependent group during the initial clinical assessment, prior to detoxification). All participants then rated current urge to drink alcohol on an 11-point anchored Likert scale ranging from 0 (“no urge to drink at all”) to 10 (“very strong urge to drink”) (Field et al., 2005).

Participants next completed the SRC task. The task comprised 3 blocks of 64 trials each. On each trial, an alcohol-related or control picture was displayed in the center of the screen, and a small manikin figure was simultaneously displayed directly above or below the picture. Each block of trials had a different stimulus-response assignment. In the “approach alcohol” block, participants were instructed to move the manikin toward alcohol-related pictures and away from stationery-related pictures. In the “avoid alcohol” block, these instructions were reversed (i.e., move the manikin away from alcohol-related pictures and toward stationery-related pictures). In the “control” block, participants were instructed to move the manikin left (or right) for alcohol-related pictures and right (or left) for alcohol-unrelated pictures. Left–right assignments were reversed for half of the participants, counterbalanced between participants within groups. Order of completion of the 3 blocks was counterbalanced between participants within groups.

In each block of the task, there were 8 practice trials, in which 4 alcohol-related and 4 control pictures were presented. Practice trials were repeated if necessary until participants understood the task requirements. This was followed by 56 test trials, split into 2 sub-blocks of 28 test trials each. Across the 56 test trials, each of the 14 alcohol-related and 14 control photographs was presented twice, once with the manikin above the photograph and once with the manikin below it. Within each block, trials were presented in a new random order for each participant. Participants were instructed to respond as quickly and accurately as possible according to the block instructions. Correct responses caused the manikin to move accordingly (toward or away from the picture, or left or right), while incorrect responses led to the presentation of a large “X” in the center of the screen, before the screen was cleared. Response accuracy and latency were recorded on each trial.

After the SRC task, participants completed the “right now” version of the AAAQ (McEvoy et al., 2004). Scores on 3 subscales were calculated: “inclined/indulgent” assessing mild approach inclinations; “obsessed/compelled” assessing intense approach inclinations; and “resolved/regulated” assessing avoidance inclinations. These subscales have high levels of internal consistency (α = 0.90, 0.86, and 0.72, respectively; McEvoy et al., 2004). Finally, participants re-rated current urge to drink alcohol, before being debriefed and given a £5 gift voucher as compensation for their time.

### Statistical Analysis

The *D*-measure scoring algorithm (Greenwald et al., 2003) was adapted to calculate 3 difference scores from the SRC task. This algorithm is recommended when there are likely between-group differences in baseline speed of responding (as was the case here: the alcohol-dependent group was significantly slower overall than the control group), and it also tends to improve correlations between automatic and self-report measures. First, response latencies faster than 300 ms or slower than 10 seconds were discarded, before mean response latency on trials when participants responded correctly was calculated for each sub-block of the “approach alcohol,”“avoid alcohol,” and “control” blocks. Next, we applied penalties for response errors by recoding response latencies on trials when participants responded incorrectly as the mean reaction time (RT) for the relevant task sub-block plus 2 standard deviations. Once error penalties had been applied, mean response latencies were recalculated for each sub-block of the approach alcohol, avoid alcohol, and control blocks. We then calculated “difference scores” for the first and second sub-blocks of each task. The “approach–avoidance” scores reflect the difference between RT in the approach alcohol block and RT in the avoid alcohol block; “approach” scores reflect the difference between the approach alcohol block and the control block; and “avoidance” scores reflect the difference between the avoid alcohol block and the control block. These difference scores for each sub-block were then divided by the pooled trials standard deviation for that sub-block (e.g., the approach–avoidance difference score derived from sub-block 1 of the approach alcohol and avoid alcohol blocks was divided by the standard deviation of RT calculated from sub-block 1 of all 3 task blocks). Finally, overall bias scores were calculated by taking the average of the bias scores from sub-block 1 and sub-block 2 (so, e.g., the “approach” bias score is simply the mean of approach bias score from sub-block 1, and approach bias score from sub-block 2) This yielded 3 “bias” scores: (i) “approach” bias score representing speed to approach alcohol-related pictures relative to baseline control condition (moving the manikin left or right), (ii) “avoidance” bias score representing speed to avoid alcohol-related pictures relative to baseline control condition, and (iii) “approach–avoidance” bias score representing speed to approach alcohol-related pictures relative to speed to avoid alcohol-related pictures. SRC task data were also analyzed using conventional scoring procedures (i.e., contrasting the raw mean RTs in different blocks), and results did not differ substantially from those reported here.

## Results

### Group Characteristics

Table 1 shows summary statistics for the alcohol-dependent and control groups. Between-group differences were explored using independent *t*-tests, or Mann–Whitney tests where variables were nonnormally distributed. As shown, groups significantly differed in age of leaving education, number of physical health conditions and prescribed medications, anxiety and depressive symptomatology on the HADS, all indices of recent alcohol consumption, severity of alcohol dependence on the LDQ, and alcohol urge measures before and after participation (all *p*s < 0.001). Groups did not differ significantly in age, *t*(125) = 0.72, ns; however, they were significantly different in gender ratio, χ^2^(1) = 5.79, *p* < 0.05.

**Table 1:** Demographic and Drinking Characteristics of Alcohol-Dependent and Control Groups

	Alcohol-dependent (*n *=* *63)		Control (*n *=* *64)				
			
	Mean	SD	Mean	SD	*t*(125)	*U*	*p*-Value
Demographic							
Age of leaving education^a^	15.79	1.04	17.32	2.41	–	1,219.00	<0.001
Physical health	3.03	1.84	0.48	0.65	–	367.50	<0.001
Medications	7.59	2.22	0.56	1.03	–	3.00	<0.001
HADS anxiety	13.89	4.13	6.78	2.83	11.31		<0.001
HADS depression	10.54	4.87	3.36	2.55	10.40		<0.001
Drinking							
Age of starting drinking^b^	16.05	3.41	16.26	1.43	–	1,645.00	ns
Weekly drinking days	6.63	0.83	1.31	1.23	–	7.50	<0.001
Total weekly units	202.23	100.03	4.35	3.85	–	0.00	<0.001
Units per drinking day	30.55	14.40	2.57	2.41	–	0.00	<0.001
Total LDQ score^c^	22.88	5.66	0.80	1.22	–	0.00	<0.001
Maximum WCAWAS score^d^	7.92	2.87	–	–	–	–	–
Urge to drink (pre)	1.93	2.21	0.70	1.30	–	1,340.00	<0.001
Urge to drink (post)	1.56	2.06	0.60	1.19	–	1,430.00	<0.001

Because of missing data ^a^*n* = 58 for clinical group, ^b^*n* = 61 for clinical group and *n *=* *63 for nonclinical group, ^c^*n* = 50 for clinical group, and ^d^*n* = 59 for clinical group (not administered to nonclinical group).

Physical health, number of current diagnosed physical health conditions; Medications, number of current prescribed medications; HADS anxiety, scores on the Hospital Anxiety and Depression Scale (HADS) anxiety subscale (possible range 0 to 21, low-high symptomatology); HADS depression, scores on the HADS depression subscale (possible range 0 to 21, low-high symptomatology); Weekly drinking days, number of days on which alcohol consumed during past week; Total weekly units, total units of alcohol consumed during past week; Units per drinking day, mean units of alcohol consumed per day when alcohol was consumed; Total LDQ score, total score on Leeds Dependence Questionnaire (LDQ) (possible range 0 to 30); Urge to drink, (possible range 0 to 10, low-high craving).

WCAWAS, Windsor Clinic Alcohol Withdrawal Assessment Scale.

### Approach and Avoidance of Alcohol Questionnaire

As predicted, the alcohol-dependent group had higher scores on the obsessed/compelled subscale (*r* = 0.58) and the resolved/regulated subscale (*r* = 0.83). Contrary to expectations, scores on the inclined/indulgent subscale (*r* = 0.25) were lower in the alcohol-dependent group, as illustrated in Table 2.

**Table 2:** Approach and Avoidance of Alcohol Questionnaire Subscale Scores in Alcohol-Dependent and Control Groups

	Alcohol-dependent (*n *=* *63)		Control (*n *=* *64)			
			
	Mean	SD	Mean	SD	*U*	*p*-Value
Inclined/indulgent	1.55	1.50	2.14	1.29	1,436.50	<0.01
Obsessed/compelled	1.36	1.38	0.11	0.24	780.50	<0.001
Resolved/regulated	5.87	1.94	0.76	1.09	107.50	<0.001

### SRC Task

All data were removed from 2 participants in the alcohol-dependent group as they were distracted mid-way through the task. A mixed 2 × 3 analysis of variance (ANOVA) with group as the between-subjects factor, and type of SRC bias score (“approach–avoidance,”“approach,”“avoidance”) as the within-subjects factor, was used to analyze the bias scores. Degrees of freedom were corrected using Greenhouse-Geisser estimates of sphericity. Contrary to predictions, the main effects of group, *F*(1, 123) = 0.88, *p* > 0.1, and the group × type of SRC bias score interaction, *F*(1.287, 148.49) = 0.72, *p* > 0.1, were not statistically significant. Table 3 shows mean SRC task bias scores, separately for the alcohol-dependent and control groups.

**Table 3:** Stimulus-Response Compatibility Task “Bias Scores” (*D-* Measures*)* in Alcohol-Dependent and Control Groups

	Alcohol-dependent (*n *=* *61)		Control (*n *=* *64)	
		
	Mean	SD	Mean	SD
Approach–avoidance	0.01	0.46	0.10	0.39
Approach (vs. control)	−1.10	0.39	−1.03	0.41
Avoidance (vs. control)	−1.11	0.35	−1.13	0.41

### Correlations Between Variables within the Alcohol-Dependent Group

Table 4 displays correlation coefficients between measures of self-reported and automatic approach and avoidance motivation, and individual differences in recent alcohol consumption. There were significant positive correlations between the AAAQ obsessed/compelled subscale (strong “approach”) and SRC task “approach” and “approach–avoidance” bias scores. There was also a nonsignificant trend for a positive correlation between the AAAQ inclined/indulgent subcale (weak “approach”) and the SRC task approach bias score. The AAAQ resolved/regulated subscale (“avoidance”) was not significantly correlated with either the “avoidance” or “approach–avoidance” SRC task bias scores, although this AAAQ subscale was significantly negatively correlated with the SRC task “approach” bias score. Individual differences in weekly alcohol consumption were positively correlated with the SRC task approach–avoidance score, and there was a trend for a positive correlation with the SRC task approach bias score. Among the control group, individual differences in weekly alcohol consumption were not significantly correlated with any of the SRC or AAAQ measures (*p*s > 0.1).

**Table 4:** Correlations Between Approach and Avoidance of Alcohol Questionnaire (AAAQ) Subscale and Stimulus-Response Compatibility (SRC) Task Difference Scores and Recent Alcohol Consumption, Among the Alcohol-Dependent Group

Variable	AAAQ I/I	AAAQ O/C	AAAQ R/R	SRC App-Av	SRC App	SRC Av
AAAQ O/C	0.58***	–				
AAAQ R/R	0.17†	0.03	–			
SRC App-Av^a^	0.07	0.31**	−0.17†	–		
SRC App^a^	0.17†	0.39**	−0.28*	0.67^a^***	–	
SRC Av^a^	0.16	0.02	−0.02	−0.57^a^***	0.23^a^*	–
Weekly alcohol consumption in U.K. units	0.02	0.07	−0.04	0.22*	0.19†	−0.06

I/I, Inclined/Indulgent; O/C, Obsessed/Compelled; R/R, Resolved/Regulated; App-Av, Approach/Avoid; App, Approach; Av, Avoid.

a Pearson’s correlations; all other correlations Spearman’s Rho correlations.

* *p *<* *0.05, **p <* *0.01, ***p <* *0.001, †*p *<* *0.10.

## Discussion

This is the first study to study the pattern of motivational conflict which is thought to operate at the levels of controlled and automatic processes among individuals with alcohol dependence. On a self-report measure, the alcohol-dependent group reported stronger intense approach and avoidance inclinations for alcohol than the control group, suggesting motivational conflict in the alcohol-dependent group and providing support for our first hypothesis. Our second hypothesis related to group differences in performance on the SRC task: We predicted that the alcohol-dependent group would show stronger automatic tendencies to both approach and avoid alcohol-related cues than the control group. However, this hypothesis was rejected, as there were no between-group differences in performance on the SRC task. However, we did find that, within the alcohol-dependent group, individual differences in the strength of automatic approach tendencies were significantly positively correlated with self-reported “approach” inclinations and negatively correlated with self-reported avoidance inclinations, which provides support for our third hypothesis. Finally, individual differences in pretreatment drinking quantity were significantly positively correlated with the strength of automatic approach tendencies in the alcohol-dependent group.

Our self-report data replicate previous results from studies that used the AAAQ: Alcohol-dependent individuals in treatment hold stronger approach and avoidance inclinations than nondependent controls (Klein et al., 2007). However, data from the SRC task suggest that alcohol-dependent patients and controls do not differ in terms of the strength of either automatic approach or avoidance responses elicited by alcohol-related cues. This stands in marked contrast to previous studies (Field et al., 2008, in press; Wiers et al., 2009) that demonstrated strong automatic approach responses among heavy drinkers who were not seeking treatment. However, we note that 1 recent study that used a similar task with alcohol-dependent inpatients also found that participants were no faster to approach alcohol-related compared to neutral pictorial cues before an experimental manipulation of approach tendencies (Wiers et al., 2011, p. 494, figure 1). However, we did find that individual differences in pretreatment drinking quantity were positively correlated with the strength of automatic approach tendencies, but not avoidance tendencies, elicited by alcohol-related cues. This raises the possibility that selection of a heavier drinking sample may have yielded the predicted group differences in performance on the SRC task. However, it is important to qualify this because drinking quantity relates to how much participants were drinking *before* they entered treatment, whereas both the AAAQ and the SRC task were measured *during* treatment.

Nonetheless, given that performance on the SRC task was correlated with pretreatment drinking quantity, but the self-report measure (the AAAQ) was not, this suggests that associations between drinking habits are automatic approach tendencies are fairly stable, but associations between drinking habits and self-reported motivational tendencies are not, with self-reports being particularly sensitive to the context and timing of assessment. It is also important to note that a causal influence of automatic approach tendencies on drinking behavior cannot be inferred from a cross-sectional study such as this one, although 2 recent studies did find evidence for a causal effect of these automatic approach tendencies on drinking behavior, in both nondependent (Wiers et al., 2010) and alcohol-dependent drinkers (Wiers et al., 2011).

The finding that automatic avoidance tendencies were comparable in alcohol-dependent and control participants is not consistent with previous reports showing that recently detoxified alcohol-dependent individuals avert their gaze from alcohol-related cues when those cues are presented for upward of 500 ms, which suggests cognitive avoidance (Noel et al., 2006; Stormark et al., 1997; Townshend and Duka, 2007). One explanation for our findings is that rapid avoidance responses to alcohol cues may not be amenable to strategic control, whereas the diversion of gaze away from alcohol-related cues may be a strategically driven process, particularly when those cues are presented for longer than a few hundred milliseconds (see Field and Cox, 2008). Alternatively, differences in the nature and treatment of the clinical sample may explain this difference, as participants in the aforementioned attentional bias studies had been abstinent for over 2 weeks and received more extensive treatment including psychotherapy, whereas our clinical group was receiving medication and nursing care only. It is therefore plausible that alcohol-dependent individuals nearing the end of detoxification, with few days of abstinence and not yet receiving psychotherapy, do not show automatic avoidance tendencies, but that these may develop with abstinence and treatment. We highlight this as a promising avenue for future research.

Our findings have a number of important theoretical implications. The profile of strong self-reported avoidance inclinations contrasts with the profile from the SRC task, as there were no significant group differences in either automatic approach or avoidance tendencies elicited by alcohol-related cues. This is inconsistent with theoretical models that propose an approach–avoidance conflict between controlled processes promoting drug avoidance and automatic processes promoting drug use (Wiers et al., 2007) and incentive-motivational theories that suggest how drug-related cues can elicit “wanting” and approach responses in the absence of awareness (Robinson and Berridge, 1993). Other motivational models of alcohol use (Cox et al., 2006) suggest that this approach–avoidance conflict exists within controlled cognitive processes (i.e., heavy drinkers will report strong desires to drink at the same time as desires to limit drinking), and our data accord with this. It was also noteworthy that our control group had higher scores on the inclined/indulgent subscale of the AAAQ (reflecting stronger mild desires and intentions to drink) than the alcohol-dependent group, although the dependent group had higher scores on the obsessed/compelled and resolved/regulated subscales (reflecting strong desires to drink, and desires to avoid drinking, respectively). This demonstrates an important qualitative distinction between mild desires to drink (which are stronger in nondependent individuals) and strong, overpowering cravings (which are stronger in dependent individuals), which suggests that the desire to drink is not a single construct that shows a linear increase as dependence progresses. Instead, alcohol dependence may involve a qualitative shift from mild desires to stronger, more obsessive cravings for alcohol.

Finally, we briefly note some limitations of our study, which primarily relate to the characteristics of the alcohol-dependent and control groups. Although groups did not differ in age, our attempts to match participants on the basis of gender ratio, educational level, and current employment status were unsuccessful. This failure to match groups on variables such as level of education should be seen in the context of previous work on cognition in substance-dependent populations, as many previous studies have been unable to match substance-dependent and control groups on this variable (e.g., Bechara et al., 2001). Future studies of this type should attempt to match alcohol-dependent and control groups on demographic and socioeconomic variables such as these, to ensure that the influence of these variables on cognitive performance can be ruled out.

To conclude, the present results help to clarify the nature of motivational conflict among alcohol-dependent individuals, and they highlight a discrepancy between self-report and automatic measures. Compared to light social drinkers, the self-reports of alcohol-dependent individuals suggest an approach–avoidance conflict; however, a measure of the automatic processing of alcohol-related cues suggests that alcohol-dependent individuals and controls do not differ on the strength of either automatic approach or avoidance tendencies elicited by alcohol-related cues.
